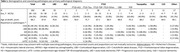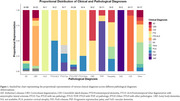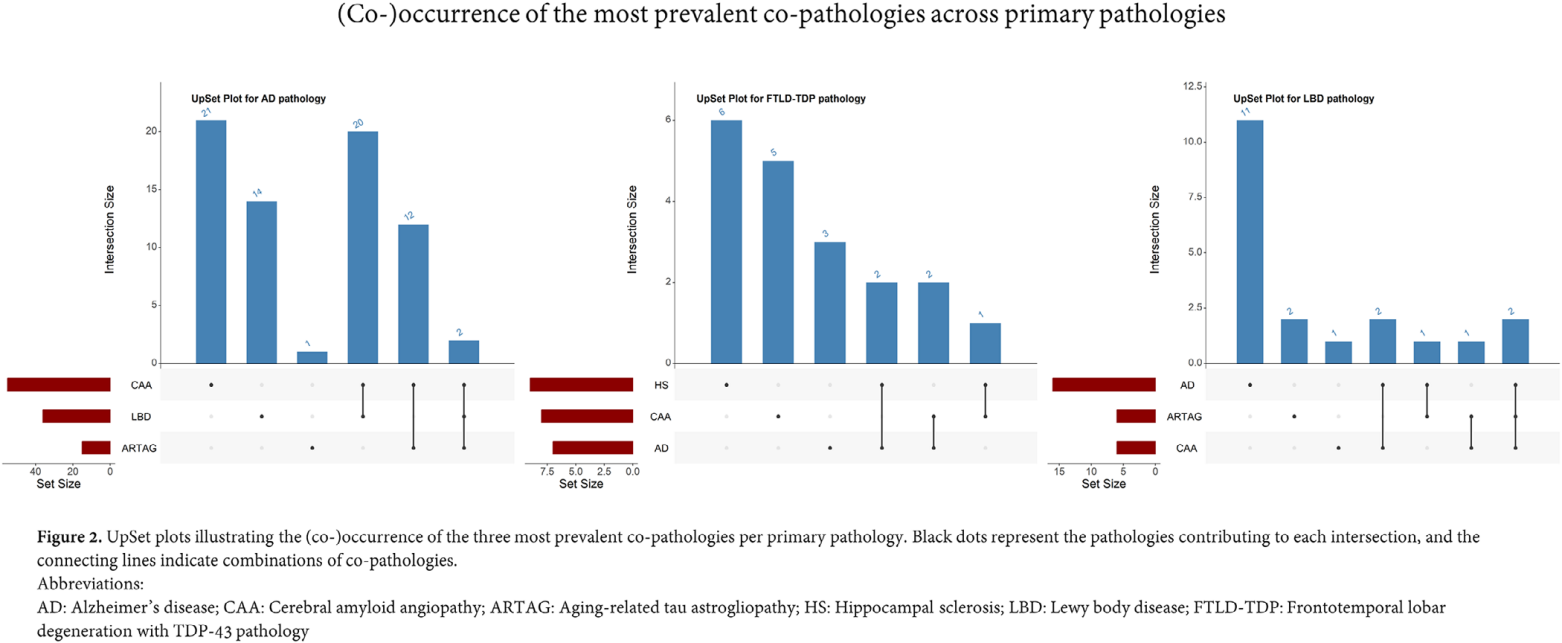# Associations between co‐pathology and diagnostic concordance in neurodegeneration: post‐mortem analysis of the Amsterdam dementia cohort

**DOI:** 10.1002/alz70855_105443

**Published:** 2025-12-24

**Authors:** Willem L. Hartog, Georgia Malliou, Michelle C. Barboure, Sanne M.M. Vermorgen, Hannah de Bruin, Argonde C. van Harten, Afina W. Lemstra, Everard G.B. Vijverberg, Wiesje M. van der Flier, Annemieke J.M. Rozemuller, Betty M. Tijms, Yolande A.L. Pijnenburg

**Affiliations:** ^1^ Alzheimer Center Amsterdam, Neurology, Vrije Universiteit Amsterdam, Amsterdam UMC location VUmc, Amsterdam, Netherlands; ^2^ Amsterdam Neuroscience, Neurodegeneration, Amsterdam, Netherlands; ^3^ Alzheimer Center Amsterdam, Department of Neurology, Amsterdam UMC, location VUmc, Amsterdam, Netherlands; ^4^ Department of Pathology, Amsterdam Neuroscience, Amsterdam UMC, Amsterdam, Netherlands; ^5^ Institute for Stroke and Dementia Research, Klinikum der Ludwig‐Maximilians Universität München, Munich, Germany; ^6^ Amsterdam Neuroscience, Neurodegeneration, Amsterdam, Noord‐Holland, Netherlands; ^7^ Neurochemistry Laboratory, Department of Clinical Chemistry, Amsterdam UMC, location VUmc, Amsterdam, Netherlands; ^8^ Department of Epidemiology and Data Science, Vrije Universiteit Amsterdam, Amsterdam UMC, Amsterdam, North Holland, Netherlands; ^9^ Alzheimer Center Amsterdam, Neurology, Amsterdam UMC Location VUmc, Vrije Universiteit Amsterdam, Amsterdam, Netherlands; ^10^ Department of Pathology, Amsterdam Neuroscience, Amsterdam UMC, Amsterdam, Noord‐Holland, Netherlands; ^11^ Alzheimer Center Amsterdam, Neurology, Vrije Universiteit Amsterdam, Amsterdam UMC location VUmc, Amsterdam, Noord‐Holland, Netherlands; ^12^ Alzheimer Center Amsterdam, Neurology, Vrije Universiteit Amsterdam, Amsterdam UMC location VUmc, Amsterdam, Amsterdam, Netherlands

## Abstract

**Background:**

Evaluation of post‐mortem brain tissue provides insight in biological heterogeneity in dementia, often showing multiple coexisting pathologies across diagnoses. Individual or combined contributions of specific (co‐)pathologies to ante‐mortem diagnostic accuracy remains largely unknown. We hypothesized that discordance between the primary pathological and clinical diagnosis is related to the presence of (multiple) co‐pathologies.

**Methods:**

We extracted clinical and pathological diagnostic data of 202 dementia patients from the Amsterdam Dementia Cohort (ADC) with available post‐mortem autopsy reports from the Netherlands Brain Bank (NBB) between 1993‐2022. Pearson chi‐square tests were performed to compare the number of co‐pathologies between diagnostically concordant and discordant cases, stratified by primary pathological diagnosis (R v.4.2.1).

**Results:**

13 primary pathological diagnoses were observed in our cohort (Table 1;Figure 1), most frequently AD (*n* = 89, 44%), FTLD‐TDP (*n* = 28, 14%), and LBD (*n* = 23, 11%). In total, 158/202 (78%) cases had concordant clinical and primary pathological diagnoses. Amongst pathological groups the highest concordance was found in AD (80/89, 90%), followed by FTLD‐TDP (25/28, 89%) and LBD (15/23, 65%). Age at death did not differ between concordant and discordant cases.

Next, we characterized the occurrence of co‐pathologies (Table 1;Figure 2), and observed ≥1 co‐pathologies most frequently in LBD cases (21/23, 91%), followed by AD (73/89, 82%), and FTLD‐TDP (22/28, 79%). In AD cases, prevalent co‐pathologies were CAA (55/89, 62%), LBD (36/89, 40%), and ARTAG (15/89, 17%). In FTLD‐TDP cases, prevalent co‐pathologies were hippocampal sclerosis (9/28, 32%), and CAA (8/28, 29%). In LBD the most frequent co‐pathology was AD (16/23, 70%). On average, the number of co‐pathologies was slightly higher in concordant compared to discordant cases, but this difference was non‐significant (*p* = 0.4). In individual pathological groups (AD, FTLD‐TDP, LBD) there was also no significant difference in the number of co‐pathologies between concordant and discordant cases (p_AD_=0.7, p_FTLD‐TDP_=0.4, p_LBD_=0.5).

**Conclusion:**

Our results highlight the pathological heterogeneity within a large memory clinic cohort, and show that neurodegenerative diseases rarely present in isolation. Although diagnostic discordance was not explained by the number of co‐pathologies or age at death, additionally investigating severity of co‐pathologies might provide insight into interactions between co‐pathologies and clinical phenotypes.